# Expression Pattern of Class B Gene *PAP3* in Flower Development of Pepper

**DOI:** 10.3390/ijms141224643

**Published:** 2013-12-18

**Authors:** Xin Li, Chen Liu, Fengjiao Da, Ning Ma, Huolin Shen

**Affiliations:** Beijing Key Laboratory of Growth and Developmental Regulation for Protected Vegetable Crops, China Agricultural University, Beijing 100193, China; E-Mails: lixin562103@163.com (X.L.); lcis250@163.com (C.L.); zhishangfengjiao@sina.com (F.D.); maning_225@163.com (N.M.)

**Keywords:** pepper, *PAP3*, expression analyses, subcellular localization

## Abstract

Class B gene *APETALA3* (*AP3*) plays a key role in the development of petals and stamens. Here, we investigated the expression pattern of *PAP3* gene (genbank accession number: HM104635) in the buds of cytoplasmic male sterility line 121A and its near-isogenic restorer line 121C at four developmental stages and analyzed the possible association between Class B genes and cytoplasmic male sterility of pepper. Semi-quantitative PCR and quantitative real-time RT-PCR (qRT-PCR) as well as RNA *in situ* hybridization showed increased expression of *PAP3* at late phase of anther development and its higher expression in restorer line compared with sterility line indicating *PAP3*’s role at late developmental stage of anther and suppressed expression in sterility line. RNA *in situ* hybridization showed Class B gene features: high abundance in stamen and petal; lower expression in pistil; no expression in sepal. Results of transient expression in onion epidermal cells also showed *PAP3* localized in the nucleus, which is consistent with the expression pattern of transcription factors of MADS-box gene family.

## 1. Introduction

Pepper (*Capsicum annuum* L.), one of the world’s most important vegetable crops with remarkable food value and economic value, is a flowering plant belonging to *Solanaceae*. Production of hybrids using cytoplasmic male sterility (CMS) represents an ideal method in seed production of crops, including pepper, due to its low cost and the high purity of seeds [1–4]. Genes of cytoplasm and nucleus regulate CMS. Regulation on the CMS associated mitochondrial genes may lead to the expression change of nuclear genes, and expression of mitochondrial sterility genes themselves can be inhibited by nuclear genes [5–7]. Currently, two CMS genes, *orf507* and *atp6-2*, were found in the pepper mitochondria [8]. As for the fertility restorer gene (*Rf*), studies reported screening using analyses based on the differenced expression but no functional genes were identified [9–11].

Developmental abnormalities of stamens may disrupt the functions of anther or pollen and eventually lead to sterility. So far, our understanding on the genetic regulation of floral organ development is largely based on the studies using dicotyledonous plants, such as *Arabidopsis*, *Antirrhinum*, *etc*. Initially, the ABC model was proposed to explain floral organ development [12], which evolved to ABCDE model [13–19]. The model defines five classes of gene function which regulate floral organ development. Among them, the function of A and E regulate the development of sepals; the function of A, B and E determine the development of petals; the function of B, C and E determine the development of stamens; the function of C and E class genes regulate the development of carpel; the function of C, D and E regulate the development of ovule [9,13,15,17–19]. As the function of B class genes regulating floral organ development, MADS-box family members *APETALA3* (*AP3*) and *PISTILLATA* (*PI*) are transcriptional factors to form *AP3*/*PI* heterodimers regulating the development of petal and stamen. Particularly, mutation of either gene may cause petals to transform sepals and stamens into carpels in some plants such as *Arabidopsis* and *Antirrhinum* [20,21]. In addition, *AP3* and *PI* regulate other genes participating in the formations of petal and stamen, while the two genes are also regulated by other genes like *LFY*, *AP1*, *UFO* and *ASK1* at different stages during flower development [22,23]. Currently, in plants, more than one *AP3*/*PI* homologous genes have been cloned and they appeared to execute different functions [24]. However, we still poorly understand their functions, respectively [25]. Though expression and function analyses of the genes may lay the foundation for revealing the stamen development process and illuminating the mechanism of male sterility, there are not any studies on peppers.

It has been shown that *AP3* gene is essential for the development of stamen in higher plants. Exogenous gene interference, silence of *AP3* and insertional mutation or deletion directional change of *AP3* may lead to the conversion of stamen to carpel at varying degrees [25–29]; no pollen production or production of infertile pollen [30,31].

Accordingly, introduction of *AP3* homolog to its mutant partially or fully restore the mutated stamens [32–34]. In addition, morphological changes may occur during development of cytoplasmic male sterile lines of these CMS model plants, such as tomatoes, carrots and tobacco [35]. These changes normally occur at the late developmental stages of the buds with the conversion of stamen to carpel [36–41]. During this process, the CMS plants show striking similarities with the changes that had been previously reported in MADS-box family B-class gene *AP3*/*PI*-like mutants of *Arabidopsis thaliana* [36,39,42]. This suggests that the regulation of B-class gene is disturbed in many CMS systems. Studies on other plant CMS systems such as wheat, the low expression level of *AP3* and *PI* genes might prevented stamens converting into pistil [43,44]. Actually, we have found the silence of *PAP3* led to the phenotype of male sterility including shriveled anthers and reduced pollen numbers in restorer line 121C using pepper as a model plant [45].

To investigate the association between the expression of *PAP3* and developmental abnormalities of anther, we analyzed the spatial and temporal expression pattern of *PAP3*, which was screened from a subtractive library of pepper, using buds of male infertility line 121A and near-isogenic restorer line 121C as test plants. This study may help us to further understand the relationship between stamen development and male sterility.

## 2. Results

### 2.1. Comparison with Anther Transcriptome

Local blast showed *PAP3* gene corresponds to comp54456_c0_seq1 in pepper anther transcriptome with a similarity of 99.85% and an *E* value of 0. There is no expression difference of comp54456_c0_seq1 between CMS line and restorer line based on the results of transcriptome sequencing.

### 2.2. Cloning of *PAP3* in CMS Line 121A

**Figure 1.**
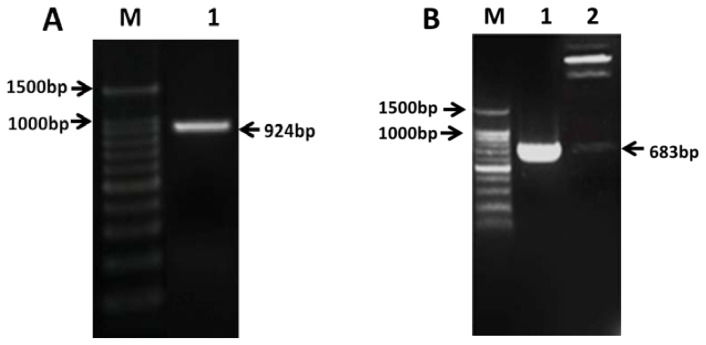
(**A**) Cloning of *PAP3* in sterile line. **M**: 100 bp DNA ladder; **1**: band of target gene (924 bp); and (**B**) Double digestion to verify the vector. **M**: 100 bp DNA ladder; **1**: recombinant vector pCAMBIA1302-*PAP3*; **2**: double digestion of pCAMBIA1302-*PAP3* at *Spe*I/*Bgl*II.

PCR amplification based on *PAP3* gene of restorer line produced 924 bp band (including ORF 681 bp) of the target gene (Figure 1A). Sequence alignment using DNAMAN version 6.0 software [46] showed no difference between the mRNA of this gene and *PAP3* gene of restorer line indicating the *PAP3* genes from the two resources are identical. Implicating the different phenotypes may result from difference of expressions instead of base sequence.

### 2.3. Construction of Transient Expressing Vector

The vector plasmid pCAMBIA1302 and target gene plasmid were digested using *Spe*I/*Bgl*II and target band was recovered to obtain the recombinant plasmid pCAMBIAl302-*PAP3* by linking vector and target gene. The recombinant plasmid was subjected to validation using PCR and enzyme digestion (Figure 1B) showing a 683 bp band, which is consistent with the inserted target gene.

### 2.4. Subcellular Localization of Gene Expression

**Figure 2.**
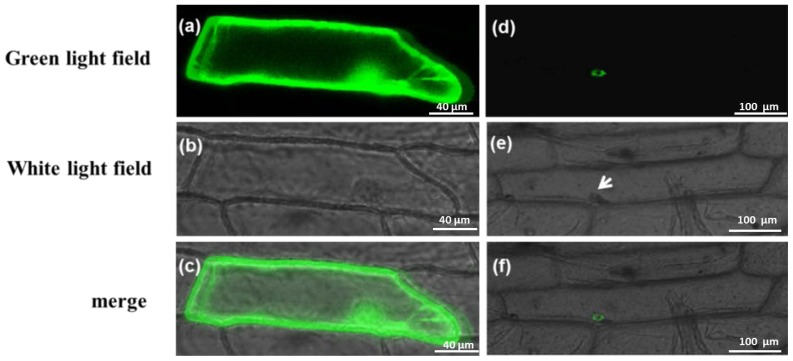
Subcellular localization of *PAP3*. (**a**–**c**) showed onion epidermal cells infected with pCAMBIA1302 vector as positive control; (**d**–**f**) showed onion epidermal cells infected with pCAMBIA1302-*PAP3*; (**a**,**d**) GFP signal; (**b**,**e**) Bright-field image; and (**c**,**f**) Merging of GFP signal, GFP signal and bright-field image. GFP-*PAP3* fusion protein was located in the nuclei. Arrows indicate nucleus. Amplification factor of the microscope was 200× (**a**–**c**) and 100× (**d**–**f**), respectively.

To investigate the subcellular distribution of *PAP3* protein in the plant, we introduced the transient expression vector pCAMBIA1302-*PAP3* fusion gene in the onion epidermal cells using gene gun bombardment and examined its expression of the green fluorescent protein (GFP) under laser confocal microscope. GFP signal could be observed throughout the cell membrane, cytoplasm and nucleus in the cells with expressing vector control pCAMBIA1302 (Figure 2c). However, GFP expression is only present in the nucleus (Figure 2f) indicating *PAP3* is a nuclear gene, a feature shared with class B transcriptional factors of MADS-box family.

### 2.5. Expression of *PAP3* Measured by Semi-Quantitative RT-PCR and qRT-PCR

**Figure 3.**
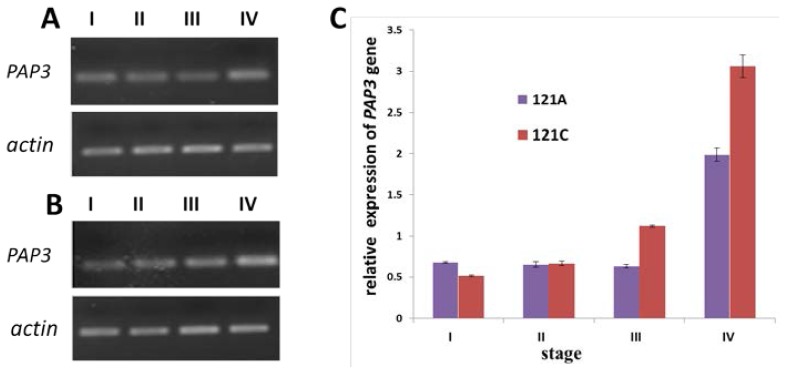
*PAP3* expression measured by semi-quantitative RT-PCR and qRT-PCR. (**A**) The expression of microspore from sterile line 121A; (**B**) the expression of microspore from restorer line 121C; and (**C**) *PAP3* expression measured by qRT-PCR. I, II, III and IV showed the four developmental phases (tetrad, early-or mid-uninucleate, late-uninucleate, binucleate) of microspore; *Actin* of Pepper served as internal control.

In order to understand the expression of *PAP3* in 121A and 121C, we initially applied RT-PCR and qRT-PCR for our research. As shown in Figure 3, *PAP3* was present in each developmental stage of CMS line and restorer line with the highest abundance in the late stage (binucleate) during microspore development (Figure 3A(IV),B(IV)). Expression level in restorer line is higher than that of CMS line (Figure 3A(IV),B(IV)).

qRT-PCR showed the similar results as semi-quantitative RT-PCR. Specifically, *PAP3* expression of restorer line in late-uninucleate and binucleate microspores was higher than that of CMS line (Figure 3C(III,IV)). However, the difference was not obvious at tetrad and early-or mid-uninucleate.

### 2.6. RNA *in Situ* Hybridization of *PAP3*

**Figure 4.**
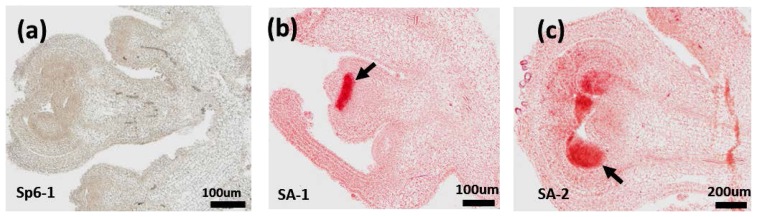
Expression of *PAP3* at apical meristem. (**b**,**c**) showed apical meristem of pepper at 20 and 25 days, respectively; and (**a**) showed SP6 negative control with no signals. Amplification factor of the microscope was 100× (**a**,**b**) and 200× (**c**), respectively. Arrows indicate sites of stamen, pistil and petal primordial with high abundance of *PAP3*.

The apical meristem picked up from the 20 and 25 day seedling of pepper was used for RNA *in situ* hybridization. In restorer line, *PAP3* is abundant in petals, stamen and pistil primordial at the early stage of flower bud differentiation (Figure 4b). Later on, *PAP3* became abundant in stamen primordial and was poorly expressed in petal primordial and pistil primordial (Figure 4c). The expression pattern of *PAP3* in 121A is similar with 121C (data not shown).

**Figure 5.**
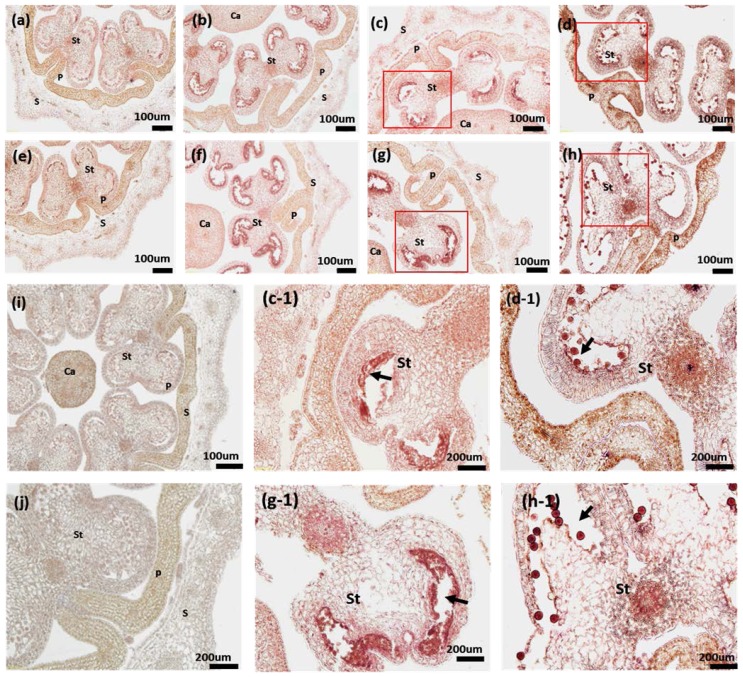
Expression of *PAP3* during bud development of pepper fertile and CMS lines. Buds from different development stages were shown under the objective of 100× (**a**–**i**) and 200× (**j**,**c**-**1**,**d**-**1**,**g**-**1**,**h**-**1**), respectively; (**i**,**j**) were SP6 negative controls; (**a**–**d**) showed the four phases during bud development (tetrad, Early-or mid-uninucleate, late-uninucleate, binucleate) in CMS line; (**e**–**h**) showed the four phases during bud development (tetrad, early-or mid-uninucleate, late-uninucleate, binucleate) in restorer line; (**c**-**1**,**d**-**1**,**g**-**1**,**h**-**1**) were picked up and amplified form (**c**,**d**,**g**,**h**), respectively. *PAP3* gene is expressed mostly in the specific organs in anther (**c**-**1**,**d**-**1**,**g**-**1**,**h**-**1**). Arrows indicate sites of anther. S, sepal; P, petal; St, stamen; Ca, carpel.

In microspores, *PAP3* mainly localizes at anthers, it expresses during the development and achieves its abundance peaks at the late stage (Figure 5). There was no obvious difference between the CMS line and restorer line at early developmental stage (tetrad and early- or mid-uninucleate) (Figure 5a,b,e,f). However, The expression of *PAP3* in restorer line is much higher than the CMS line (Figure 5c-1,d-1,g-1,h-1) at late developmental stage (late-uninucleate, binucleate). These results suggest *PAP3* gene may be involved in the regulation of pollen development, especially the mature process of pollen.

## 3. Discussion

In most studied angiosperms, *AP3* and *PI* genes were shown to be expressed in petals and stamens except that they are occasionally present in the first and fourth whorls of flower and non-floral tissues [47]. The class B gene expressed in the developmental petals and stamens of *Brassica napus* L. *AP3* and *PI* expressed in floral tissue of *Arabidopsis* and seeds, leaves and roots of maize [48–50]. Class B genes were also shown to expressed in vascular bundle, stalk, embryonic primordial of developing tubes in aconite (*Eranthis hyemalis*) [51]. These studies suggest expression patterns of class B genes such as *AP3* and *PI* are not conservative and vary in different plants [52].

*PAP3* expression sites of flower are similar in CMS line and restorer line. *PAP3* is abundant in stamen but not in petal primordia at the early stage of flower bud development indicating *PAP3* may regulate the development of petal and stamen. Besides, *PAP3* is present in stamen, pistil and petals through the bud development with the highest abundance in stamen. However, *PAP3* is not expressed in sepals. Our findings is consistent with early report showing continuous expression of developmental marker gene like *PAP3* not only occurs in primordial of specific flora organ but also continues to the late stage of development [53].

Prior studies have shown that expression of *AP3* and *PI* genes are suppressed in the flowers of sterile plants [42,44,54]. Other studies showed distinct expression levels of *AP3* gene between sterile and fertile lines and its abundance in sterile line was lower than in fertile line at the late bud development stage [36,43,55]. In the present study, we found *PAP3* expression is low at the early stage during microspore development and increased at late stage in both CMS line and restorer line indicating *PAP3* is not only present in floral primordia but also may play a role in the pollen maturation process. In addition, *PAP3* showed similar expression levels at early stages during microspore development between the CMS and restorer lines but expression of CMS line became much lower than the restorer line at late developmental stage. But *PAP3*’s counterpart unigene comp54456_c0_seq1 in pepper transcriptome showed similar expression levels in CMS and restorer lines, which could be explained by the sequencing of transcriptome using mixed anthers from different developmental stages [43].

During anther development, abnormity in any stage may affect the normal development of pollen microspore. We found pollen from male sterile line showed irregular shape, uneven size and emptiness and spallation at the late anther developmental stage (Figure 5d-1). However, restorer line appeared uniformed size, plump wall and free of shrinkage (Figure 5h-1) with lower expression level of CMS *PAP3* compared with restorer line at late developmental stage of anther. Thus, the morphological difference may suggest *PAP3* plays a role in the anther development, which warrants further studies for validation.

## 4. Experimental Section

### 4.1. Materials of Plants

Cytoplasmic male sterility line 121A and its isogenic restorer 121C were cultivated in the greenhouse of experimental station in China Agricultural University in 2012. Buds from the four developmental phases (tetrad, early-or mid-uninucleate, late-uninucleate, binucleate) in bud stage [56] were used for *in situ* hybridization and collected anther was used for semi-quantitative RT-PCR and qRT-PCR. Anther was harvested from the buds of 121A and 121C with white petals and used to clone *PAP3* gene and analyze transient expression in onion epidermal cells. In addition, the apical meristem of the seedling was harvested from 20d and 25d cultivation of CMS line and restorer lines for *in situ* hybridization.

### 4.2. Gene Cloning and Blast

An EST showing 91% homology with class B gene *TAP3* in Tomato flower was identified by screening using cDNA library induced by pepper CMS, which was constructed previously in our lab. We obtained 929 bp full length gene by RACE technology and named it as *PAP3* (genbank accession number: HM104635). Phylogenetic analysis showed *PAP3* is clustered into one group with the *AP3* gene of *Arabidopsis* [45].

Blasting *PAP3* in pepper anther transcriptome which was established in our lab [4] (with the same lines) was performed to identify the sequence with the highest similarity.

### 4.3. RNA Extraction and cDNA Synthesis

RNA was isolated using SV total RNA Isolation System Kit (Promega Inc., Madison, WI, USA) following instructions. cDNA was synthesized using PrimeScript 1st Strand cDNA synthesis kit (Takara, Dalian, China).

### 4.4. Cloning of *PAP3* in CMS Line

*PAP3* gene in CMS line was cloned used primers F and R (Table 1) designed based on *PAP3* full length sequence. PCR was performed in total volumes of 25 μL containing 1 μL of cDNA, 5 μL of 5× PrimeSTAR^®^ Buffer (Mg^2+^ plus; Takara, Dalian, China), 15.75 μL of ddH_2_O, 2 μL of dNTP mixture, 0.5 μL of specific F/R primers respectively, 0.25 μL of PrimeSTAR^®^ HS DNA Polymerase. The PCR condition was as follows: 94 °C for 3 min; 35 cycles of 94 °C for 40 s, 55 °C 40 s, 72 °C for 1 min; finally 72 °C for 8 min. The product was separated on 1% agarose gel electrophoresis and purified using a DNA purification kit (BIOMED, Beijing, China) then sequenced. The clone and sequence were repeated 20 times.

### 4.5. Transient Expression of *PAP3* in Onion Epidermal Cells

Based on the full length *PAP3* sequence and pCAMBIA1302 vector’s restriction sites, two enzyme restrictions sites *Spe*I and *Bgl*II were picked up to design the primers SL-F and SL-R (Table 1 underlines indicate digestion sites of *Spe*I and *Bgl*II). Reverse transcription cDNA was used as template to amplify the coding region of the target gene (stop code was not included). The resulting PCR amplified products were inserted to the pCAMBIA1302 vector at the *N*-terminus of the GFP gene to generate pEGFP-*PAP3*. Verified by sequencing, pEGFP-*PAP3* and an empty vector were transferred into onion epidermal cells using the particle bombardment method, respectively. The fluorescence signals were detected using laser confocal microscope.

### 4.6. Semi Quantitative RT-PCR and qRT-PCR

The pepper actin (GenBank: GQ339766.1) gene was served as the internal control of semi-quantittive RT-PCR and qRT-PCR. The cycling parameters of relative RT-PCR were: 94 °C for 3 min followed by 28 cycles of 94 °C for 30 s, 53 °C for 30 s, 72 °C for 30 s, and final elongation at 72 °C for 3 min. PCR products were visualized by 1% gel electrophoresis. qRT-PCR was performed using THUNDERBIRD SYBR qPCR Mix From BEIJING TINYOO Biotechnology Co., Ltd (Beijing, China). The primers for semi-quantitive RT-PCR and qRT-PCR were listed in Table 1 (*PAP3*-F and *PAP3*-R). Expression levels of the unigenes were calculated from the threshold cycle using the 2^−ΔΔ^*^CT^* method [57].

### 4.7. *In Situ* Hybrization

Specific primers SH-F and SH-R were designed according to *PAP3* gene (Table 1) to prepare probe template (product contains ORF excluded stop code). Digoxigenin-labeled sense and antisense probes of *PAP3* gene were generated using SP6/T7 RNA polymerase through PCR amplification of cDNA and then kept in 50% formamide. Fixation of the samples and paraffin sectioning were previously described.

Before the hybridization, the sections were pretreated (dewaxing, rehydration and protease treatment). The glycine buffer was used to stop the reaction and the tissue was re-fixed. After acetic anhydride treatment following washing and dehydration, the class was kept in sealed plastic boxes at 4 °C for 4–5 h. The diluted probes were denatured at 80 °C for 2 min and kept on ice.

*In situ* hybridization was performed following a protocol described elsewhere [58].

## 5. Conclusions

Through expression analyses we confirmed the *PAP3* gene as a class B gene of pepper, for its location in nucleus and highest expression in stamen. Our results also showed significantly higher expression in 121C than 121A during late-uninucleate and binucleate phases of microspore. Though preliminary functional verification by virus induced gene silencing has been implemented previously, transgenic experiments still need to be done for further verification of *PAP3* gene for its effect on another development in the pepper cytoplasmic male sterile line.

## Figures and Tables

**Table 1.** Primers for expression analyses of *PAP3*.

Primers	Sequences (5′–3′)
F	AGACCTTTTAGGGTTTGAGT
R	ACACACTGAATTAAGCAAAA
PAP3-F	GGTGGATTAGTTGAGCAGGA
PAP3-R	GATGATTTGGTTGAAGGCGT
ACTIN-F	AGCACCTCTCAACCCTAA
ACTIN-R	GCAAAGCATAACCCTCAT
SH-F	GATTTAGGTGACACTATAGAATGCTAGA
	AAATAGAAAAAAAGTATGGCTC
SH-R	TGTAATACGACTCACTATAGGG
	ACCTAGACCAAAAGTAGTAATATCA
SL-F	GAAGATCTTCAGAAAATAGAAAAAAAGTATGGCTC
SL-R	GGACTAGTCC ACCTAGACCAAAAGTAGTAATATCA
